# A filtering strategy identifies FOXQ1 as a potential effector of lamin A dysfunction

**DOI:** 10.18632/aging.100483

**Published:** 2012-08-31

**Authors:** Jose Candelario, Leng-Ying Chen, Paul Marjoram, Sita Reddy, Lucio Comai

**Affiliations:** ^1^ Department of Molecular Microbiology & Immunology, University of Southern California, Los Angeles, CA 90033 USA; ^2^ Department of Molecular Biology & Biochemistry, University of Southern California, Los Angeles, CA 90033 USA; ^3^ Department of Preventive Medicine, University of Southern California, Los Angeles, CA 90033 USA; ^4^ Institute for Genetic Medicine, Keck School of Medicine, University of Southern California, Los Angeles, CA 90033 USA; ^5^ La Jolla Bioengineering Institute, San Diego, CA 9212

**Keywords:** health care, diseases, aging, rapamycin

## Abstract

Small increases in the expression of wild-type prelamin A are sufficient to recapitulate the reduced cell proliferation and altered nuclear membrane morphology observed in cells expressing progerin, the mutant lamin A associated with progeria. We hypothesized that the manifestation of these phenotypes in cells expressing elevated levels of wild-type prelamin A or progerin is caused by the same molecular effectors, which play a central role in the onset of the progeroid phenotype. To experimentally test this hypothesis, we compared the transcriptomes of isogenic diploid fibroblasts expressing progerin or elevated levels of wild-type prelamin A with that of wild-type fibroblasts. We subsequently used the reversion towards normal of two phenotypes, reduced cell growth and dismorphic nuclei, by treatment with farnesyltransferase inhibitor (FTI) or overexpression of ZMPSTE24, as a filtering strategy to identify genes linked to the onset of these two phenotypes. Through this analysis we identified the gene encoding for the transcription factor FOXQ1, as a gene whose expression is induced in both cells expressing progerin and elevated levels of wild-type prelamin A, and subsequently reduced in both cell types upon conditions that ameliorate the phenotypes. We overexpressed FOXQ1 in normal fibroblasts and demonstrated that increased levels of this factor lead to the development of both features that were used in the filtering strategy. These findings suggest a potential link between this transcription factor and cell dysfunction induced by altered prelamin A metabolism.

## INTRODUCTION

Lamin A is a component of the nuclear lamina that is synthesized as a prelamin A precursor that then undergoes several sequential post-translational modifications to generate mature lamin A. Mutations in the lamin A/C gene are associated with a variety of disorders, collectively termed laminopathies [1], among which is the progeroid disease Hutchinson-Gilford Progeria Syndrome (HGPS) [2]. HGPS (progeria) and Werner syndrome, also known as adult progeria, are genetic diseases that may provide insights on the mechanisms of normal human aging [3, 4]. The majority of cases of HGPS are caused by a point mutation in the lamin A/C gene that lead to the production of a permanently farnesylated mutant lamin A protein termed progerin [5, 6]. The production of progerin leads to the progressive appearance of several cellular alterations that cause the onset of an accelerated aging phenotype. Progeria cells and HeLa cells expressing ectopic progerin display growth defects and altered nuclear membrane morphology. Both cellular phenotypes can be improved by inhibition of farnesylation by treatment with farnesyl transferase inhibitors (FTIs) [7-11]. More recently, several studies have demonstrated that treatment with rapamycin, an inhibitor of the growth-promoting mTOR signaling pathway and potential drug for anti-aging therapy [12], limits the accumulation of progerin, improves the cellular phenotype of progerin-expressing cells and rescue tissue functions in lamins deficient mice [13-16]. These findings suggest that mammalian target of rapamycin (mTOR) inhibitors could be of therapeutic value for progeria and possibly other laminopathies [17].

We and others have shown that small increases in the expression of wild-type prelamin A are sufficient to recapitulate the cellular defects observed in HGPS cells including dismophic nuclei and reduced cell proliferation resulting from increased incidence of apoptosis and premature senescence [11, 18]. Cells expressing progerin or elevated levels of wild-type prelamin A show abnormal lamin A aggregates in the nucleus, which are also observed in cells from normal old-age individuals but absent in cells from young-age individuals [11]. Although the relationship between HGPS and the onset of similar phenotypes during the lifespan of a normal individual is not know, these findings suggest an intriguing link between dysfunctional prelamin A processing and normal aging [19]. Indeed, other studies have suggested that alterations in the lamin A pathway may play a key role in general human aging [20-22].

Lamin A is believed to provide a mechanical framework for the support of the nuclear envelope [23, 24] and influence the spatial organization of chromosomes and chromatin structure, ultimately affecting gene expression [1, 23, 25, 26]. Accumulation of progerin has been shown to cause a decrease in the levels of heterochromatin and abnormal telomeres length homeostasis [18, 20, 27-29], and alters genes expression [30-33]. However, a direct relationship between altered gene expression and the phenotypic changes of cells expressing dysfunctional prelamin A has not been established.

## RESULTS

### Identification of changes in gene expression in cells expressing progerin or elevated levels of prelamin A

**Figure 1:**
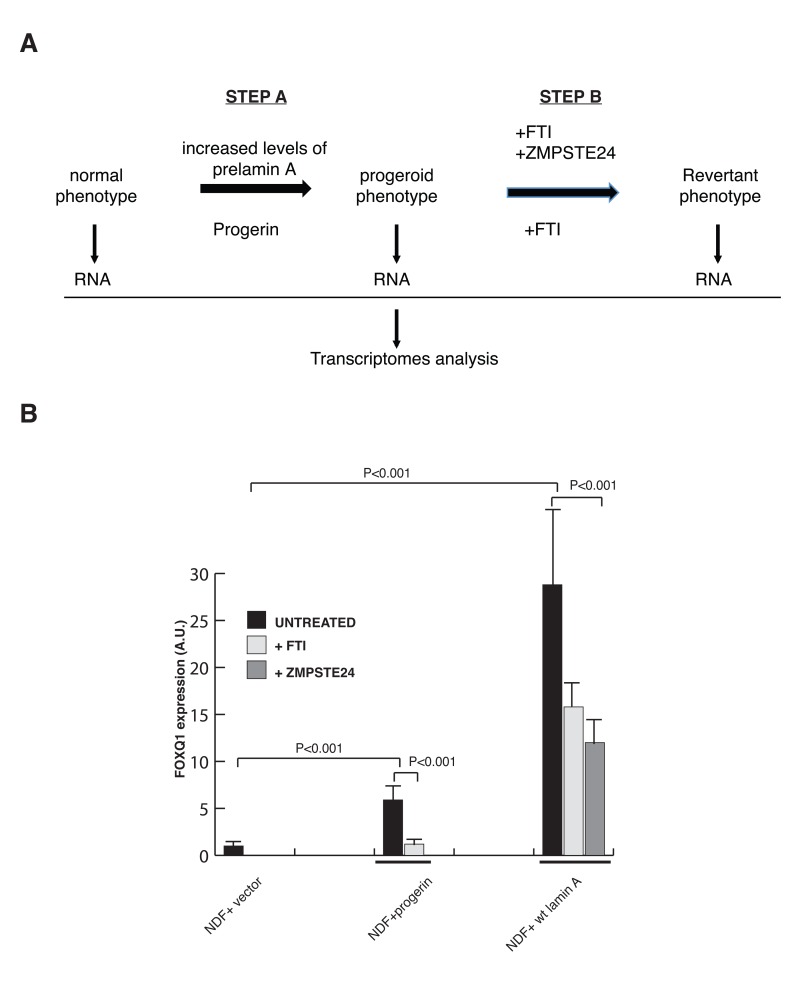
A filtering strategy to identify effectors of lamin A dysfunction (**A**) Strategy utilized to identify FOXQ1 as a gene whose expression is altered in cells expressing progerin or elevated levels of prelamin A and reversed towards normal after treatment with FTIs or ZMPSTE24 overexpression. (**B**) Quantitative RT-PCR analysis of FOXQ1 expression in the indicated fibroblast lines confirms that FOXQ1 expression is upregulated in cells expressing elevated levels of prelamin A or progerin, and reverted towards normal after treatment with FTI or overexpression of ZMPSTE24.

We have previously demonstrated that treatment with FTI or increased expression of ZMPSTE24, a key prelamin A processing enzyme, improve cell proliferation and lead to a significant decrease in the number of cells with dismophic nuclei in cells expressing elevated levels of prelamin A [11], indicating that abnormal prelamin A processing is responsible for these phenotypic changes. Since expression of progerin or increased expression of prelamin A leads to remarkably similar cellular phenotypes, we reasoned that the cellular alterations observed in these cells are likely caused by shared molecular effectors that play a significant role in inducing the progeroid phenotype. To test experimentally this hypothesis, we carried out microarray analyses of gene expression profiling in isogenic normal human diploid fibroblast lines to identify genes whose expression pattern is altered upon expression of either progerin or elevated levels of wild-type prelamin A (Figure 1, Step A). This analysis demonstrates changes in the expression (>2 fold; p<0.05) of more than 1800 genes in cells with progerin or elevated levels of prelamin A (Figure S1A). To define how these changes in gene expression may influence cell homeostasis, we analyzed our microarray data with Database for Annotation, Visualization, and Integrated Discovery (DAVID) software, and identified enrichment in genes linked to many biological pathways (Figures S1B). Significantly, several of the pathways, including the extracellular matrix (ECM)-receptor interaction and WNT signaling, which have previously been implicated in the pathology of progeria [33], were shared between cells expressing progerin or elevated levels of prelamin A (Figure S1C).

### Identification of genes that are reverted by treatment with farnesyl transferase inhibitor (FTI) and ZMPSTE24 overexpression

A filtering strategy was then used to identify potential key effectors of lamin A dysfunction (Figure 1A, step B). Specifically, we searched for genes whose expression reverted towards normal after treatment of both cell lines with farnesyl transferase inhibitors (FTIs) as well as, in cells with elevated levels of wild-type prelamin A, after ZMPSTE24 overexpression (Figures S2 and S3). Through this analysis we identified one gene, which encodes for the forkhead transcription factor FOXQ1, as the only gene whose expression was induced in cells expressing progerin and cells with elevated levels of wild-type prelamin A, and subsequently reduced toward normal in both cell types upon treatments that ameliorates the defects in growth and nuclear membrane morphology. The differential expression of FOXQ1 was validated by quantitative reverse-transcriptase polymerase chain reaction (RT-qPCR) analysis (Figure 1B). FOXQ1 is a poorly characterized member of the forkhead family of transcription factors that has been implicated in the regulation of epithelial cell morphology and differentiation [34, 35].

### Ectopic expression of FOXQ1 in human diploid fibroblasts impairs proliferation and results in dysmorphic nuclei

**Figure 2:**
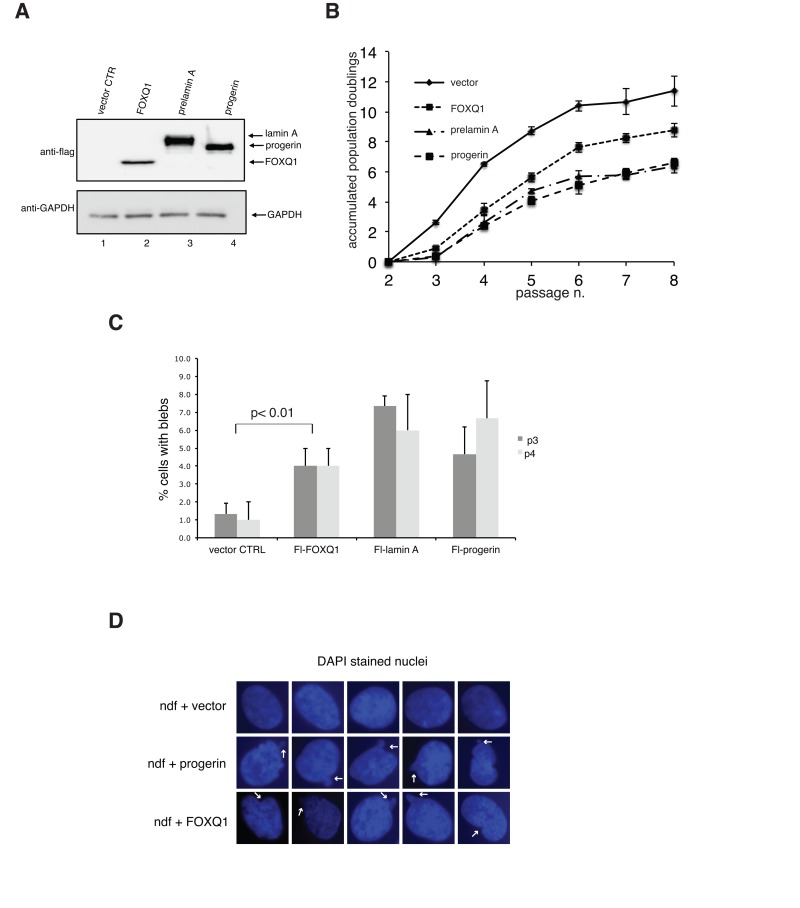
Ectopic expression of FOXQ1 in normal human diploid fibroblasts results in reduced growth rates and altered nuclear membrane morphology, two phenotypes that are observed in cells expressing progerin or elevated levels of prelamin A (**A**) Human diploid fibroblasts expressing flag-tagged FOXQ1, progerin or prelamin A were lysed and analyzed by immunoblotting with flag antibodies. Antibodies against glyceraldehyde 3-phosphate dehydrogenase (GAPDH) were used as loading control. (**B**) Normal human diploid fibroblasts were transduced with lentiviruses for the expression of flag-tagged FOXQ1, progerin or prelamin A and cell growth was monitored over several passages. Control cell line represents human diploid fibroblasts transduced with a lentivirus for the expression of GFP. (**C**) Percentage of cells with dysmorphic nuclei at passage 3 and 4 in each experimental sample was determined as described in the materials and methods section. (**D**) Representative images DAPI stained nuclei in control cells and cells overexpressing progerin or FOXQ1 showing altered nuclear morphology. Arrows point to examples of nuclear blebbing.

To define the functional link between FOXQ1 and the development of progeroid features induced by dysfunctional prelamin A processing, we overexpressed FOXQ1 in normal human diploid fibroblasts and monitored both growth and nuclear morphology of cells in culture over several passages (Figure 2). Strikingly, we noted that increased expression of FOXQ1 is accompanied by reduced rates of proliferation and by a significant number of cells with dysmorphic nuclei after a few passages compared to control cells (Figure 2B-D). Importantly, these changes are qualitatively and quantitatively similar to those observed in progeria cells, cells ectopically expressing progerin and cells with elevated levels of prelamin A (Figure 2) [11]. Growth inhibition is restricted to primary cells, since expression of FOXQ1 as well as progerin does not affect growth of transformed cells including HeLa and HEK293 (data not shown). The presence of nuclear blebbing in cells over-expressing FOXQ1 is remarkable, as this is a prominent feature of cells with altered lamin A function [19].

## DISCUSSION

The poor proliferation of fibroblasts expressing progerin is the result of both premature senescence and apoptosis [11], two key features of cells expressing progerin or elevated levels of prelamin A that are likely to contribute to the premature aging phenotype of HGPS. How accumulation of progerin or partially processed prelamin A results in the premature onset of cell dysfunction is unknown but likely linked to alterations in fundamental nuclear processes.

Here, we implemented a stringent screen to identify genes whose altered expression could contribute to the onset of the defective phenotypes induced by dysfunctional prelamin A processing. To this end, we compared the transcriptome of isogenic fibroblasts expressing either the mutant form of lamin A associated with progeria (termed progerin) or elevated levels of wild-type prelamin A, with that of wild-type fibroblasts. We subsequently used the reversion of two phenotypes, cell proliferation and nuclear membrane morphology, towards normal by treatment with farnesyltransferase inhibitors (FTI) and ZMPSTE24 overexpression as a filtering strategy to identify key downstream effectors. This analysis identified FOXQ1, a subtelomeric gene that encodes for a forkhead transcription factor, as the only statistically significant gene whose expression is induced in both cells expressing progerin and cells with elevated levels of wild-type prelamin A, subsequently reduced in both cell types upon treatment with FTI as well as in cells expressing elevated levels of lamin A after ZMPSTE24 overexpression. Strikingly, ectopic expression of FOXQ1 in normal human fibroblasts leads to the development of both features that were used in the filtering strategy (growth defects and alterations in nuclear membrane morphology) in a qualitative and quantitative manner similar to that observed in cells with dysfunctional prelamin A processing. However, we did not observe a significant improvement in growth nor nuclear membrane morphology in progerin-expressing cells after downregulation of FOXQ1 by siRNAs (data not shown), suggesting that altered expression of additional genes contributes to the maintenance of these phenotypes.

Although there is no prior link between FOXQ1 and aging-associated pathologies, there have been studies suggesting that FOXOs, a subfamily of the forkhead transcription factors, play a protective role against age-associated diseases including diabetes, cancer, autoimmune syndromes and neurodegeneration [36]. FOXOs inhibit mTOR [37, 38], a potential substrate for targeted therapy [17]; thus manipulation of FOXOs function could provide clinical benefit for HGPS. Whether FOXQ1 impacts mTOR function remains to be determined.

Lamin A interacts with chromosomes and accumulation of progerin has been shown to cause a decrease in the level of peripheral heterochromatin, loss of heterochromatin associated with the inactive X chromosome [27, 28], relocalization or decrease levels of markers of heterochromatin including heterochromatin protein 1a (HP1a), histone H3 trimethylated on lysine 9 (H3K9-3me) and histone H3 trimethylated on lysine 27 (H3K27-3me) [20, 28, 39, 40]. These findings suggest that epigenetic alterations caused by progerin may induce changes in the expression of specific genes, which contribute to the onset of the defective phenotypes. These epigenetic changes could be limited to a gene promoter or, since lamins have been implicated in the positioning of chromosomal domains within the nucleus [41], comprise large chromosomal regions as a consequence of altered spatial distribution of chromosomes. Indeed, comparative genomic hybridization studies have shown that disruption of lamins function differentially influence the relocalization of chromosomes to nuclear “blebbed” sites [42]. Interestingly, the gene encoding for FOXQ1 is located on the subtelomeric domain of chromosome arm 6p, a chromosome arm that was consistently overrepresented in nuclear membrane blebs induced by lamin deficiency [42].

Changes in gene expression between cells from HGPS patients and normal controls have been reported [30-32]. However, very little overlap in the identity of differentially expressed genes have been observed, possibly suggesting that genetic heterogeneity in the cell lines used for these studies influenced the outcome of the analyses. Alternatively, progerin may induce stochastic epigenetic alterations that affect distinct gene sets, each of which contributes in distinct ways to the onset of the progeroid phenotypes.

## METHODS

### Cell culture

Primary dermal fibroblast cell lines from healthy newborn (GM00038 and AG12945), individuals were obtained from the Coriell Cell Repository. HeLa and HEK293 cells were obtained from ATCC (CRL-1573). Cells were grown in Dulbecco's modified Eagle's medium (DMEM) supplemented with 15% fetal bovine serum, 2 mM L-glutamine, 100 U mL^−1^ penicillin and 100 μg mL–1 streptomycin at 37 °C in 5% CO2, and 3% O2. Cells seeded at 1.4 × 105 per 100-mm-diameter dish were passaged when cultures reached 85% confluency. Cell growth was measured by calculation of accumulated population doublings using the formula (log *H* − log *S*)/ log 2, where log *H* is the logarithm of the number of cells harvested and log *S* is the logarithm of the number of cells seeded on the first day of each passage, as described in [11]. Treatment of fibroblast lines with FTI and ZMPSTE24 overexpression were carried out as described in [11].

### RNA isolation

Total RNA was isolated from each fibroblast line at passage 10 using RNeasy kit from QIAGEN according to the manufacture's protocol and quantitated by assessing absorbance at 260 and 280 nm using a NanoDropTM 1000 spectrophotometer. Three micrograms of total RNA was then submitted to the University of Southern California Affymetrix MicroArray Core Facility at Children's Hospital Los Angeles for processing, chip hybridization, and scanning. Gene expression was analyzed on an Affimetrix gene chip Human Genome U133 Plus 2.0 Array, which offers comprehensive genome wide expression on a single array with over 47,000 transcripts and variants, including 38,500 well characterized genes. A Fluidics Station 400 (Affymetrix) was used to wash and stain the chips and fluorescence was detected using a G2500 GeneArray Scanner (Hewlett-Packard).

### Microarray Data analysis

Raw data were analyzed initially using Microarray Suite version 5.0 (MAS 5.0, Affymetrix), which was used for quality control analysis, to scale all values to a target value (250), and to generate a list of ‘absent’ genes. Arrays were judged as acceptable for additional analysis if the 3'/5' ratio of GAPDH and β-actin was less than 3, and the percentage of genes found to be ‘present’ was similar from array to array. Low-level analysis (background correction, normalization, and gene summarization) of microarray data was performed with Microarray Suite 5.0 (MAS 5.0). Individual arrays were analyzed and scaled with MAS 5.0 using manufacturer's default thresholds for detection calls to attain intensity signals, detection p-value, and signal log ratio. Detection of significantly differentially expressed genes between Affymetrix GeneChips was attained using the Significance-Score (S-score) algorithm (Bioconductor; http://biocondctor.org). S-scores p-values of 0.01 were used as the threshold. P-values higher than 0.01 between the Affymetrix GeneChips were filtered out and were not included for the subsequent analysis. Gene lists were attained using Microsoft Excel to filter for differences between arrays with significant p-values according to fold changes and to uncover genes that were significantly reverted. Microarray experiments conform to the MIAME guidelines and a complete data set has been submitted to the National Center for Biotechnology Information (NCBI) Gene Expression Omnibus database (GEO).

### Heat Maps

Gene Cluster 3.0 software, developed by Michael Eisen at Stanford University (http//bonsai.ims.u-tokyo.ac.jp/%7Emdehoon/software/cluster/software.htm) was used to cluster the gene list attained from filtering according to gene expression similarity and function. The output of Cluster 3.0 was then imported in Java Tree View [43] to generate heatmap images.

### Pathways analysis

Database for Annotation, Visualization and Integrated Discovery (DAVID) software (http://david.abcc.ncifcrf.gov) was utilized to compare co-expression interactions with interaction information that was manually curated from the literature and to annotate these interactions with the closest matching biological functions. This software package utilizes information derived from the literature to identify functional relationships between genes and various biological processes and molecular functions.

### Quantitative RT-PCR

Quantitative reverse transcription PCR (qPCR) was performed using the BIORAD iCycler instrument. RNA from each cell line was extracted and purified using the RNeasy kit (Qiagen, Valencia, CA, USA) according to the manufacturer's instructions. For each sample, 3 μg of RNA were transcribed using the first strand cDNA synthesis kit from Amersham Biosciences for 1 h at 37 °C, after 10 min denaturation at 65 °C. Primers for specific detection of FOXQ1 were: (FOXQ1-428F: 5'-CGGAGATCAACGAGTACCTCA -3'; FOXQ1-591R: 5'-GTTGAGCATCCAGTAGTTGTCCTT-3'). The glyceroldehyde 3-phosphate dehydrogenase gene (GAPDH) was used as the internal standard. Primers for (GAPDH) were used for normalization (GAPDH-F: 5'-CCACCCATGGCAAATTCCATG-3'; GAPDH-R:5'-TGATGGGATTTCCATTGATGAC-3'). PCR products were separated on 2% agarose gels and stained with Ethidium Bromide. iQ SYBR Green was used for real-time PCR along with the MyiQ software according to the manufacture's instructions. All PCR efficiencies were > 95% and were determined for each primer set using a standard curve.

### Plasmids and generation of stably transduced cell lines

Human FOXQ1 cDNA was purchased from ATCC (10436949) and cloned into the pCR4-topo vector by PCR using the following primers: 5'-CATATGAAGT: TGGAGGTGTTCGTC-3', 5'-TCTAGATCAGGCTAC GAGCGTCTC-3'. Sequence accuracy was verified by DNA sequencing. The FOXQ1 cDNA was subcloned into the Nde1–EcoR1 sites of the pVL1393-Flag vector (Comai et al., 1994). Flag-tagged FOXQ1 cDNA was then subcloned into the *BamHI/XbaI* sites of the lentiviral transfer vector pkey204 MSH2-IRES-GFP to generate pkey-FlagFOXQ1. Progerin, prelamin A, and FOXQ1 were also subcloned into the *Nde1/EcoR1* sites of the CMV promoter containing lentiviral vector pRRL.sin.CMV.flag.sv40.puromycin. Recombinant lentiviruses were generated as previously described [44]. For lentiviral infection, calcium phosphate transfected 293T cell cultures were trypsinized, seeded onto 100-mm plates, and incubated at 37°C for 24 h. The supernatant containing viral particles was collected, filtered and equal volumes of each viral supernatant were added to normal human fibroblast cultures that were ~40% confluent. After 6 h incubation at 37°C, the supernatant was removed, the cells were washed twice with phosphate buffered saline (PBS) and incubated in DMEM containing 10% serum at 37°C. Transduced cells expressing the GFP were selected by fluorescence-activated cell sorting. The expression of flag-tagged proteins was analyzed by immunoblotting with flag antibodies (Sigma, St. Louis, MO, USA). Each lentivirus was transduced in two independent fibroblast cell lines and each cell line was grown in duplicate.

### Nuclear morphology analysis

Nuclear morphology was analyzed by fluorescence microscopy of DAPI-stained cells. At each passage, 2.3 × 10^3^ cells were seeded in chambered slides and analyzed. Cells were washed in PBS and fixed using 4% paraformaldehyde for 5 min at room temperature. Fixed cells were then washed again in PBS and incubated in a permeabilization solution (0.1% Triton X-100, 0.1% sodium citrate) for 5 min on ice. Cells were then washed with PBS and DAPI (1 μg mL^−1^ in PBS) was added for 1 minute in the dark. Subsequently the cells were washed in a permeabilization solution followed by PBS to remove the detergent. Each slide was then treated with antifade reagent and allowed to dry in the dark. Nuclear morphology was analyzed with a fluorescence microscope at x200, x400, and x1000 magnification. Nuclei with blebs were considered as those with one or more lobulations resulting in misshaped nuclei. At each passage, two independent observers scored 300 cells for each cell line.

### Western blot analysis

Human fibroblasts were washed twice in PBS, collected, and lysed in SDS Sample Buffer at 95 °C for 5 min. Cell extracts were resolved by SDS-PAGE and transferred to polyvinylidene fluoride (PVDF) membranes. Blots were probed with anti-FOXQ1 (goat polyclonal; Santa Cruz Biotechnology, Santa Cruz, CA, USA, sc-47596), anti-GAPDH (goat polyclonal; Santa Cruz Biotechnology, Santa Cruz, CA, USA, sc-20357), and anti-Flag (mouse monoclonal, Sigma, F-3165). Immunoreactive bands were detected with the appropriate horseradish peroxidase-conjugated secondary antibodies (Pierce, Rockford, IL, USA) and visualized by enhanced chemiluminescence (Amersham, Piscataway, NY, USA).

### Statistical analysis

We performed statistical analysis of differences between the cell lines and GFP control using the Student's *t*-test. We used Microsoft Excel for calculations of Student's t-test and standard deviations.

## SUPPLEMENTARY FIGURES



## References

[R1] Broers JL, Ramaekers FC, Bonne G, Yaou RB, Hutchison CJ (2006). Nuclear lamins: laminopathies and their role in premature ageing. Physiol Rev.

[R2] Ackerman J, Gilbert-Barness E (2002). Hutchinson-Gilford progeria syndrome: a pathologic study. Pediatr Pathol Mol Med.

[R3] Dreesen O, Stewart CL (2011). Accelerated aging syndromes, are they relevant to normal human aging?. Aging.

[R4] Li B, Jog S, Candelario J, Reddy S, Comai L (2009). Altered nuclear functions in progeroid syndromes: a paradigm for aging research. TheScientificWorldJournal.

[R5] De Sandre-Giovannoli A, Bernard R, Cau P, Navarro C, Amiel J, Boccaccio I, Lyonnet S, Stewart CL, Munnich A, Le Merrer M, Levy N (2003). Lamin a truncation in Hutchinson-Gilford progeria. Science.

[R6] Eriksson M, Brown WT, Gordon LB, Glynn MW, Singer J, Scott L, Erdos MR, Robbins CM, Moses TY, Berglund P, Dutra A, Pak E, Durkin S (2003). Recurrent de novo point mutations in lamin A cause Hutchinson-Gilford progeria syndrome. Nature.

[R7] Capell BC, Erdos MR, Madigan JP, Fiordalisi JJ, Varga R, Conneely KN, Gordon LB, Der CJ, Cox AD, Collins FS (2005). Inhibiting farnesylation of progerin prevents the characteristic nuclear blebbing of Hutchinson-Gilford progeria syndrome. Proceedings of the National Academy of Sciences of the United States of America.

[R8] Glynn MW, Glover TW (2005). Incomplete processing of mutant lamin A in Hutchinson-Gilford progeria leads to nuclear abnormalities, which are reversed by farnesyltransferase inhibition. Human molecular genetics.

[R9] Mallampalli MP, Huyer G, Bendale P, Gelb MH, Michaelis S (2005). Inhibiting farnesylation reverses the nuclear morphology defect in a HeLa cell model for Hutchinson-Gilford progeria syndrome. Proceedings of the National Academy of Sciences of the United States of America.

[R10] Toth JI, Yang SH, Qiao X, Beigneux AP, Gelb MH, Moulson CL, Miner JH, Young SG, Fong LG (2005). Blocking protein farnesyltransferase improves nuclear shape in fibroblasts from humans with progeroid syndromes. Proceedings of the National Academy of Sciences of the United States of America.

[R11] Candelario J, Sudhakar S, Navarro S, Reddy S, Comai L (2008). Perturbation of wild-type lamin A metabolism results in a progeroid phenotype. Aging Cell.

[R12] Blagosklonny MV (2010). Rapamycin and quasi-programmed aging: four years later. Cell Cycle.

[R13] Ramos FJ, Chen SC, Garelick MG, Dai DF, Liao CY, Schreiber KH, Mackay VL, An EH, Strong R, Ladiges WC, Rabinovitch PS, Kaeberlein M, Kennedy BK (2012). Rapamycin Reverses Elevated mTORC1 Signaling in Lamin A/C-Deficient Mice, Rescues Cardiac and Skeletal Muscle Function, and Extends Survival. Science translational medicine.

[R14] Cao K, Graziotto JJ, Blair CD, Mazzulli JR, Erdos MR, Krainc D, Collins FS (2011). Rapamycin reverses cellular phenotypes and enhances mutant protein clearance in Hutchinson-Gilford progeria syndrome cells. Science translational medicine.

[R15] Cenni V, Capanni C, Columbaro M, Ortolani M, D'Apice MR, Novelli G, Fini M, Marmiroli S, Scarano E, Maraldi NM, Squarzoni S, Prencipe S, Lattanzi G (2011). Autophagic degradation of farnesylated prelamin A as a therapeutic approach to lamin-linked progeria. European journal of histochemistry: EJH.

[R16] Choi JC, Muchir A, Wu W, Iwata S, Homma S, Morrow JP, Worman HJ (2012). Temsirolimus activates autophagy and ameliorates cardiomyopathy caused by lamin a/c gene mutation. Science translational medicine.

[R17] Blagosklonny MV (2011). Progeria, rapamycin and normal aging: recent breakthrough. Aging.

[R18] Huang S, Risques RA, Martin GM, Rabinovitch PS, Oshima J (2008). Accelerated telomere shortening and replicative senescence in human fibroblasts overexpressing mutant and wild-type lamin A. Experimental cell research.

[R19] Reddy S, Comai L (2012). Lamin A, farnesylation and aging. Experimental cell research.

[R20] Scaffidi P, Misteli T (2006). Lamin A-dependent nuclear defects in human aging. Science.

[R21] McClintock D, Ratner D, Lokuge M, Owens DM, Gordon LB, Collins FS, Djabali K (2007). The Mutant Form of Lamin A that Causes Hutchinson-Gilford Progeria Is a Biomarker of Cellular Aging in Human Skin. PLoS ONE.

[R22] Ragnauth CD, Warren DT, Liu Y, McNair R, Tajsic T, Figg N, Shroff R, Skepper J, Shanahan CM (2010). Prelamin A acts to accelerate smooth muscle cell senescence and is a novel biomarker of human vascular aging. Circulation.

[R23] Goldman RD, Gruenbaum Y, Moir RD, Shumaker DK, Spann TP (2002). Nuclear lamins: building blocks of nuclear architecture. Genes Dev.

[R24] Holmer L, Worman HJ (2001). Inner nuclear membrane proteins: functions and targeting. Cell Mol Life Sci.

[R25] Han X, Feng X, Rattner JB, Smith H, Bose P, Suzuki K, Soliman MA, Scott MS, Burke BE, Riabowol K (2008). Tethering by lamin A stabilizes and targets the ING1 tumour suppressor. Nat Cell Biol.

[R26] Zastrow MS, Vlcek S, Wilson KL (2004). Proteins that bind A-type lamins: integrating isolated clues. J Cell Sci.

[R27] Goldman RD, Shumaker DK, Erdos MR, Eriksson M, Goldman AE, Gordon LB, Gruenbaum Y, Khuon S, Mendez M, Varga R, Collins FS (2004). Accumulation of mutant lamin A causes progressive changes in nuclear architecture in Hutchinson-Gilford progeria syndrome. Proceedings of the National Academy of Sciences of the United States of America.

[R28] Shumaker DK, Dechat T, Kohlmaier A, Adam SA, Bozovsky MR, Erdos MR, Eriksson M, Goldman AE, Khuon S, Collins FS, Jenuwein T, Goldman RD (2006). Mutant nuclear lamin A leads to progressive alterations of epigenetic control in premature aging. Proceedings of the National Academy of Sciences of the United States of America.

[R29] Decker ML, Chavez E, Vulto I, Lansdorp PM (2009). Telomere length in Hutchinson-Gilford progeria syndrome. Mech Ageing Dev.

[R30] Csoka AB, English SB, Simkevich CP, Ginzinger DG, Butte AJ, Schatten GP, Rothman FG, Sedivy JM (2004). Genome-scale expression profiling of Hutchinson-Gilford progeria syndrome reveals widespread transcriptional misregulation leading to mesodermal/mesenchymal defects and accelerated atherosclerosis. Aging Cell.

[R31] Scaffidi P, Misteli T (2008). Lamin A-dependent misregulation of adult stem cells associated with accelerated ageing. Nat Cell Biol.

[R32] Marji J, O'Donoghue SI, McClintock D, Satagopam VP, Schneider R, Ratner D, Worman HJ, Gordon LB, Djabali K (2010). Defective lamin A-Rb signaling in Hutchinson-Gilford Progeria Syndrome and reversal by farnesyltransferase inhibition. PLoS One.

[R33] Hernandez L, Roux KJ, Wong ES, Mounkes LC, Mutalif R, Navasankari R, Rai B, Cool S, Jeong JW, Wang H, Lee HS, Kozlov S, Grunert M (2010). Functional coupling between the extracellular matrix and nuclear lamina by Wnt signaling in progeria. Developmental cell.

[R34] Bieller A, Pasche B, Frank S, Glaser B, Kunz J, Witt K, Zoll B (2001). Isolation and characterization of the human forkhead gene FOXQ1. DNA Cell Biol.

[R35] Feuerborn A, Srivastava PK, Kuffer S, Grandy WA, Sijmonsma TP, Gretz N, Brors B, Grone HJ (2010). The Forkhead factor FoxQ1 influences epithelial differentiation. J Cell Physiol.

[R36] Salih DA, Brunet A (2008). FoxO transcription factors in the maintenance of cellular homeostasis during aging. Current opinion in cell biology.

[R37] Chen CC, Jeon SM, Bhaskar PT, Nogueira V, Sundararajan D, Tonic I, Park Y, Hay N (2010). FoxOs inhibit mTORC1 and activate Akt by inducing the expression of Sestrin3 and Rictor. Developmental cell.

[R38] Southgate RJ, Neill B, Prelovsek O, El-Osta A, Kamei Y, Miura S, Ezaki O, McLoughlin TJ, Zhang W, Unterman TG, Febbraio MA (2007). FOXO1 regulates the expression of 4E-BP1 and inhibits mTOR signaling in mammalian skeletal muscle. J Biol Chem.

[R39] Scaffidi P, Misteli T (2005). Reversal of the cellular phenotype in the premature aging disease Hutchinson-Gilford progeria syndrome. Nat Med.

[R40] Columbaro M, Capanni C, Mattioli E, Novelli G, Parnaik VK, Squarzoni S, Maraldi NM, Lattanzi G (2005). Rescue of heterochromatin organization in Hutchinson-Gilford progeria by drug treatment. Cell Mol Life Sci.

[R41] Mewborn SK, Puckelwartz MJ, Abuisneineh F, Fahrenbach JP, Zhang Y, MacLeod H, Dellefave L, Pytel P, Selig S, Labno CM, Reddy K, Singh H, McNally E (2010). Altered chromosomal positioning, compaction, and gene expression with a lamin A/C gene mutation. PLoS One.

[R42] Shimi T, Pfleghaar K, Kojima S, Pack CG, Solovei I, Goldman AE, Adam SA, Shumaker DK, Kinjo M, Cremer T, Goldman RD (2008). The A- and B-type nuclear lamin networks: microdomains involved in chromatin organization and transcription. Genes Dev.

[R43] Saldanha AJ (2004). Java Treeview--extensible visualization of microarray data. Bioinformatics.

[R44] Li B, Navarro S, Kasahara N, Comai L (2004). Identification and biochemical characterization of a Werner's syndrome protein complex with Ku70/80 and poly(ADP-ribose) polymerase-1. J Biol Chem.

