# Supplementary material for: A filtering strategy identifies FOXQ1 as a potential effector of lamin A dysfunction
Source: Aging (Albany NY). 2012 Aug 31;4(8):567–77. doi: 10.18632/aging.100483 (PMC3461344; doi:10.18632/aging.100483)
Supplement: Supplementary file 1 [file aging-04-567-s001.pdf]

mTOR signaling in mammalian skeletal muscle. J Biol Chem. 2007; 282:21176-21186.
39. Scaffidi P and Misteli T. Reversal of the cellular phenotype in the premature aging disease Hutchinson-Gilford progeria syndrome. Nat Med. 2005; 11:718-719.
40. Columbaro M, Capanni C, Mattioli E, Novelli G, Parnaik VK, Squarzoni S, Maraldi NM and Lattanzi G. Rescue of heterochromatin organization in Hutchinson-Gilford progeria by drug treatment. Cell Mol Life Sci. 2005; 314:453-562.
41. Mewborn SK, Puckelwartz MJ, Abuisneineh F, Fahrenbach JP, Zhang Y, MacLeod H, Dellefave L, Pytel P, Selig S, Labno CM, Reddy K, Singh H and McNally E. Altered chromosomal positioning, compaction, and gene expression with a lamin A/C gene mutation. PLoS One. 2010; 5:e14342.
42. Shimi T, Pfleghaar K, Kojima S, Pack CG, Solovei I, Goldman AE, Adam SA, Shumaker DK, Kinjo M, Cremer T and Goldman RD. The A- and B-type nuclear lamin networks: microdomains involved in chromatin organization and transcription. Genes Dev. 2008; 22:3409-3421.
43. Saldanha AJ. Java Treeview--extensible visualization of microarray data. Bioinformatics. 2004; 20:3246-3248.
44. Li B, Navarro S, Kasahara N and Comai L. Identification and biochemical characterization of a Werner's syndrome protein complex with Ku70/80 and poly(ADP-ribose) polymerase-1. J Biol Chem. 2004; 279:13659-13667.

SUPPLEMENTARY FIGURES

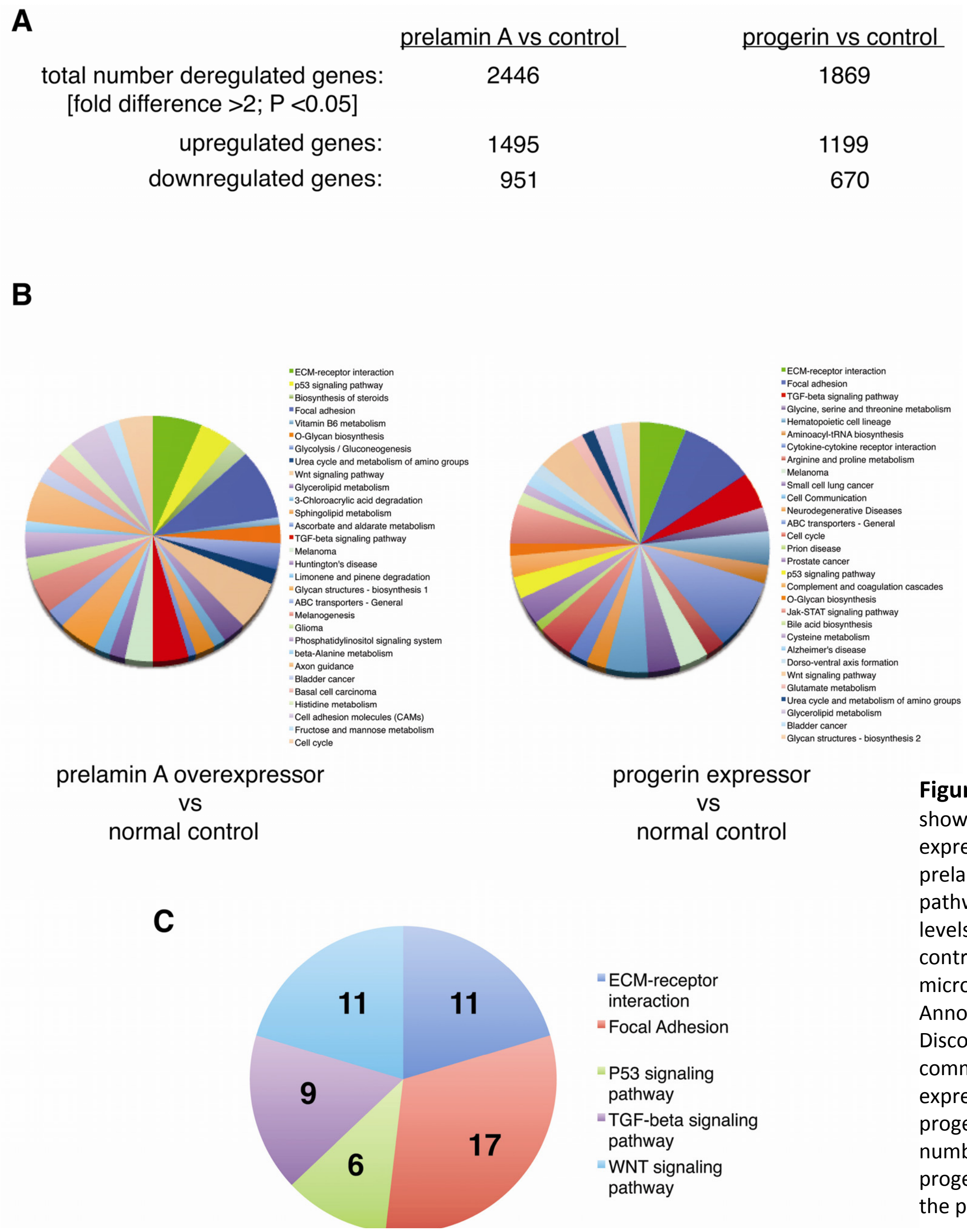

Figure S1. (**A**) Summary of microarray data showing number of genes with altered expression in cells expressing elevated levels of prelamin A or progerin. (**B**) Pie charts showing pathways altered in cells expressing elevated levels of prelamin A or progerin, as compared to control cells. Chart was generated from the DNA microarray data analysis by Database for Annotation, Visualization, and Integrated Discovery (DAVID). (**C**) Pie chart showing common pathways affected in both cells expressing elevated levels of prelamin A or progerin. Numbers in each sector represent number of deregulated genes shared between progerin and elevated levels of prelamin A within the pathway.

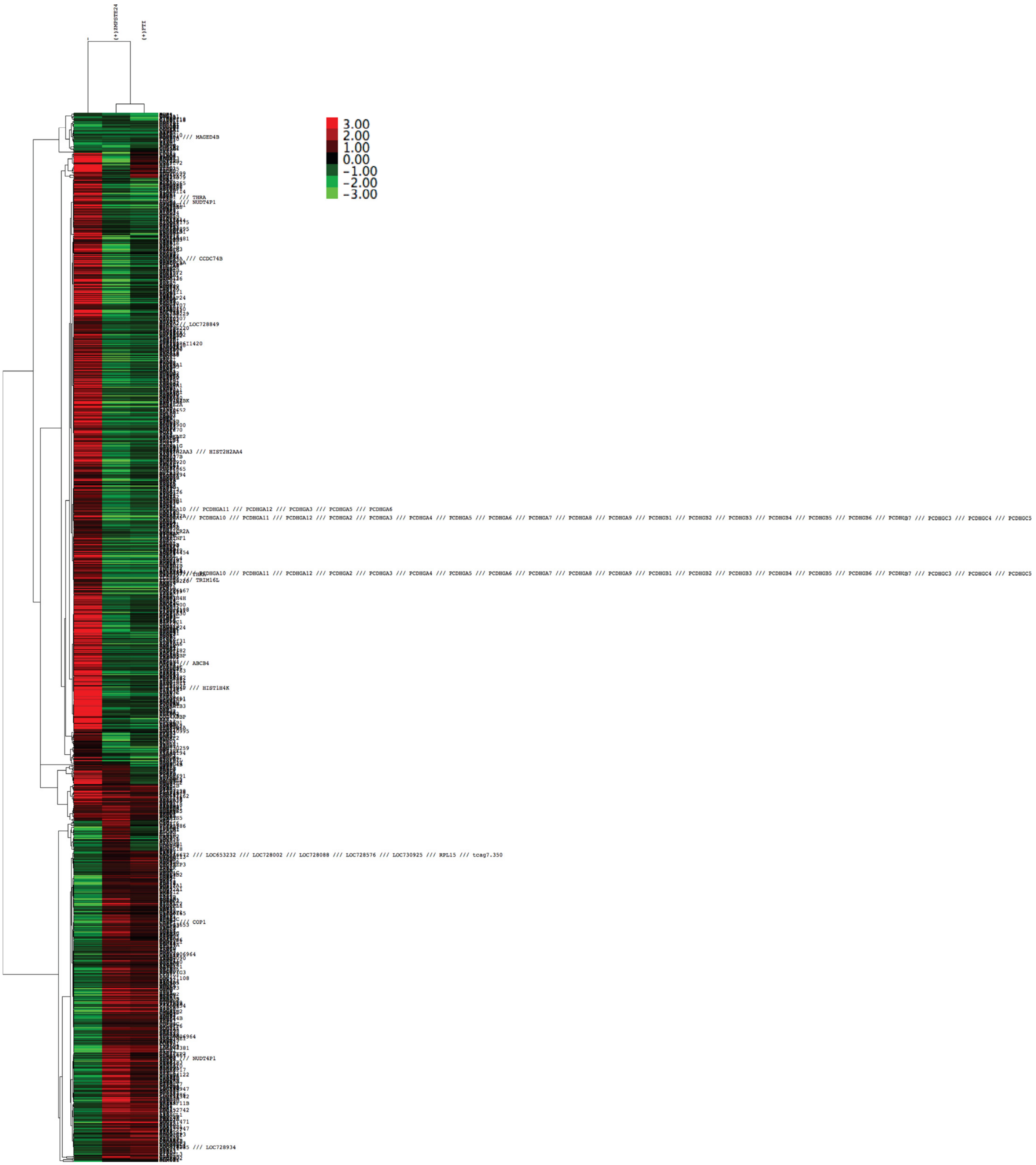

Figure S2. Unsupervised hierarchical clustering of differentially expressed genes from DNA microarray data analysis showing gene expression changes in cells expressing elevated levels of prelamin A prior to and after FTI treatment for 48 hours or ZMPSTE24 overexpression compared to control cells. Genes that are up- or down-regulated by ≥2-fold with $p<0.05$ were subjected to centered Pearson's correlation matrix. Each heat map represents intensity values relative to the median intensity across all samples per probe after background subtraction and normalization.

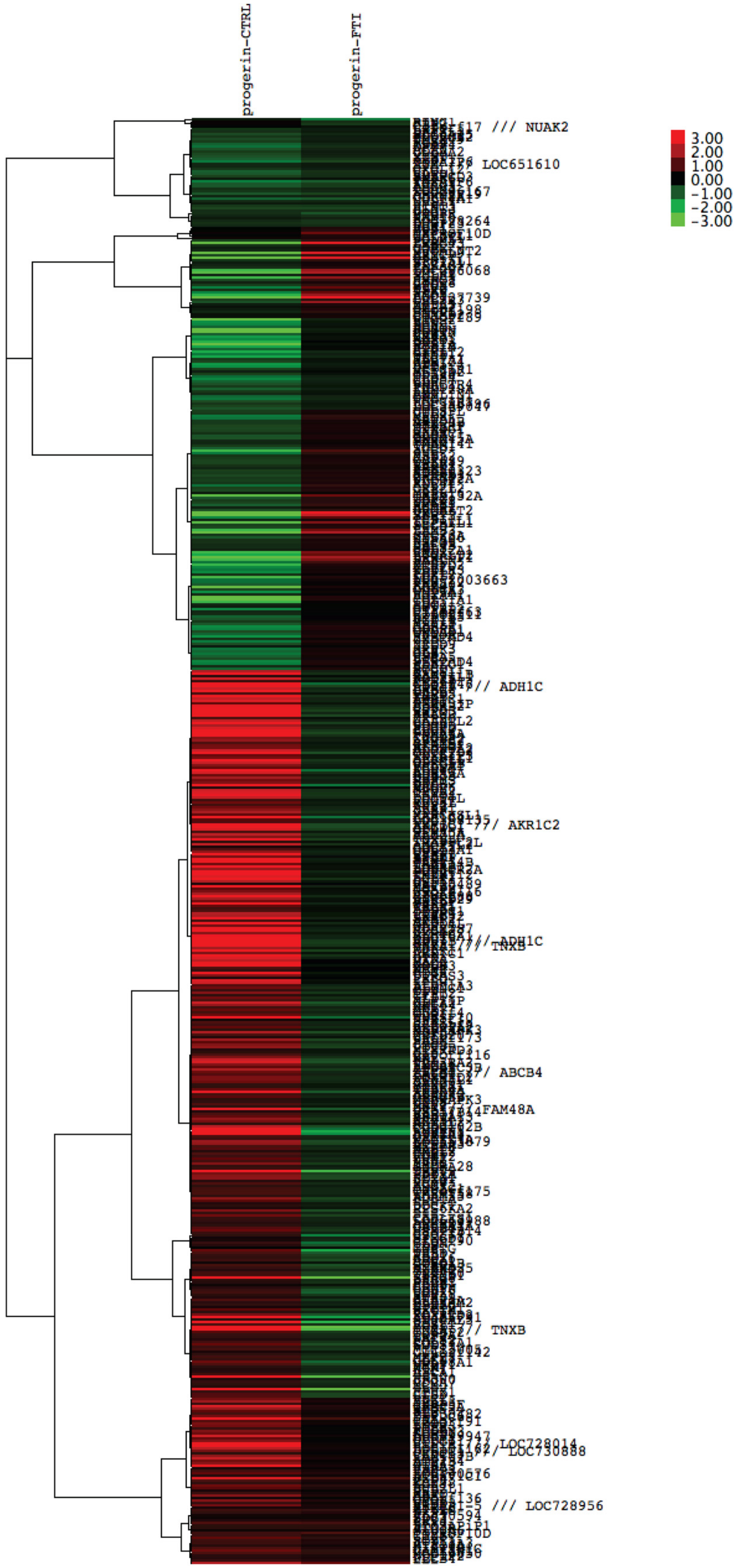

Figure S3. Unsupervised hierarchical clustering of differentially expressed genes from DNA microarray data analysis showing gene expression changes in cells expressing progerin prior to and after FTI treatment compared to control cells. Genes that are up- or down-regulated by ≥2-fold with p<0.05 were subjected to centered Pearson's correlation matrix. Each heat map represents intensity values relative to the median intensity across all samples per probe after background subtraction and normalization.
